# Primary tracheal schwannoma a review of a rare entity: current understanding of management and followup

**DOI:** 10.1186/s13019-017-0677-2

**Published:** 2017-11-28

**Authors:** Shadi Hamouri, Nathan M. Novotny

**Affiliations:** 1 Department of General Surgery and Urology, Jordan University of Science and Technology, Faculty of Medicine, King Abdullah University Hospital, Irbid, 22110 Jordan; 2 Department of Surgery, Oakland University William Beaumont School of Medicine, Beaumont Health, 3535 W 13 Mile Rd. Ste 307, Royal Oak, MI 48126 USA

**Keywords:** Trachea, Neurogenic tumors, Schwannoma, Endoscopic resection, Recurrence, Surgical resection

## Abstract

**Background:**

Neurogenic tumors of the tracheobronchial tree are extremely rare and include neurofibroma and schwannoma. We report a case of primary recurrent tracheal schwannoma causing obstructive airway symptoms.

**Case presentation:**

A 60-year-old man presented with obstructive airway symptoms due to recurrent tracheal schwannoma. Due the recurrence, size of the tumor and low surgical risk, the patient was treated with tracheal resection.

**Conclusion:**

Primary endotracheal neurogenic tumors are extremely rare, but one should consider them in the differential diagnosis of persistent upper airway symptoms. While endoscopic therapies recur nearly a quarter of the time, surgical resections do not have any recorded recurrences.

## Background

Primary tracheal tumors are rare. Together, malignant squamous cell and adenoid cystic carcinoma compose around 75% of the tumors. The other quarter of the tumors is composed of multiple histological subtypes that include benign, intermediate, and malignant tumors [1]. Neurogenic tumors of the tracheobronchial tree are extremely rare and include neurofibroma and schwannoma [1, 2]. We report a case of primary recurrent tracheal schwannoma causing obstructive airway symptoms.

## Case presentation

**Fig. 1:**
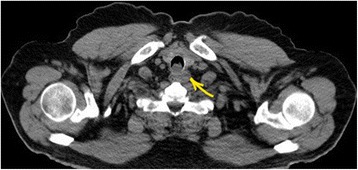
CT scan of the chest showing dumbbell shape endotracheal tumor with extratracheal extension

**Fig. 2:**
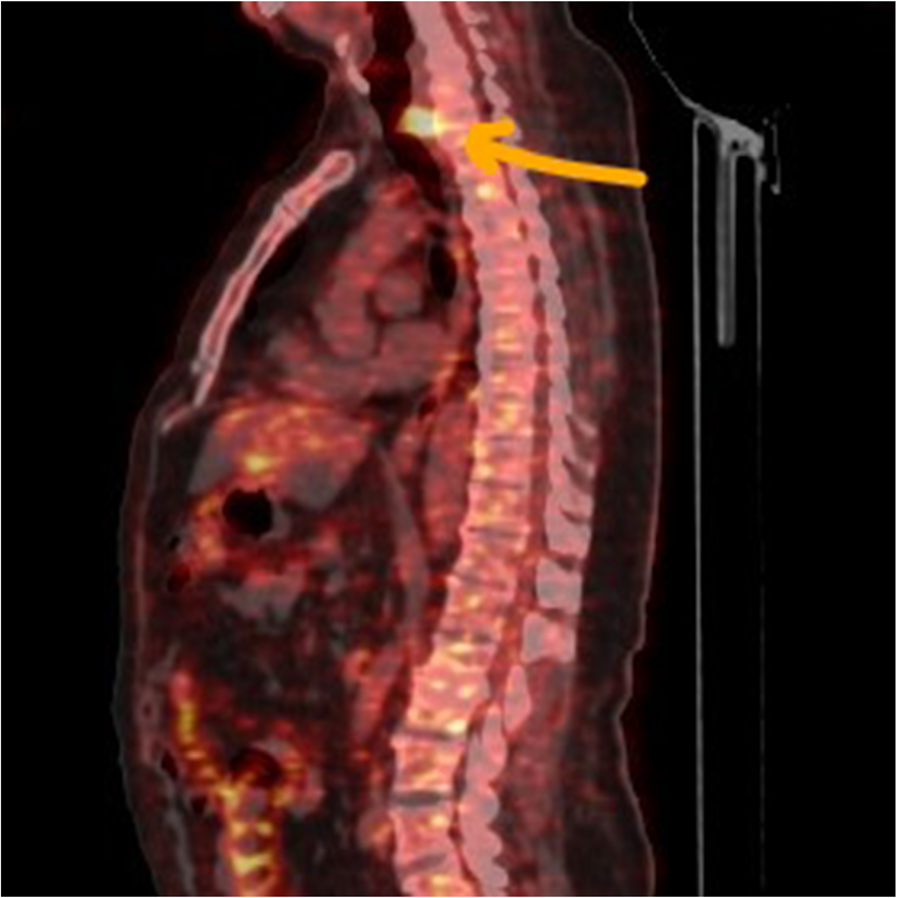
PET-CT sagittal view showing hyperactive lesion with an SUV of 6.5

**Fig. 3:**
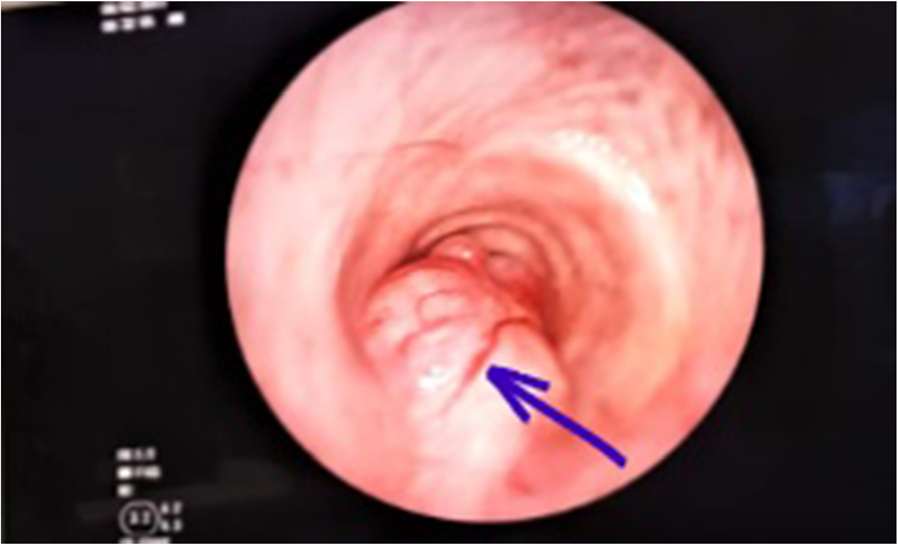
Bronchoscopic view. The upper border of the lesion is 3 cm from the vocal cords. The arrow points to the small superficial blood vessels

**Fig. 4:**
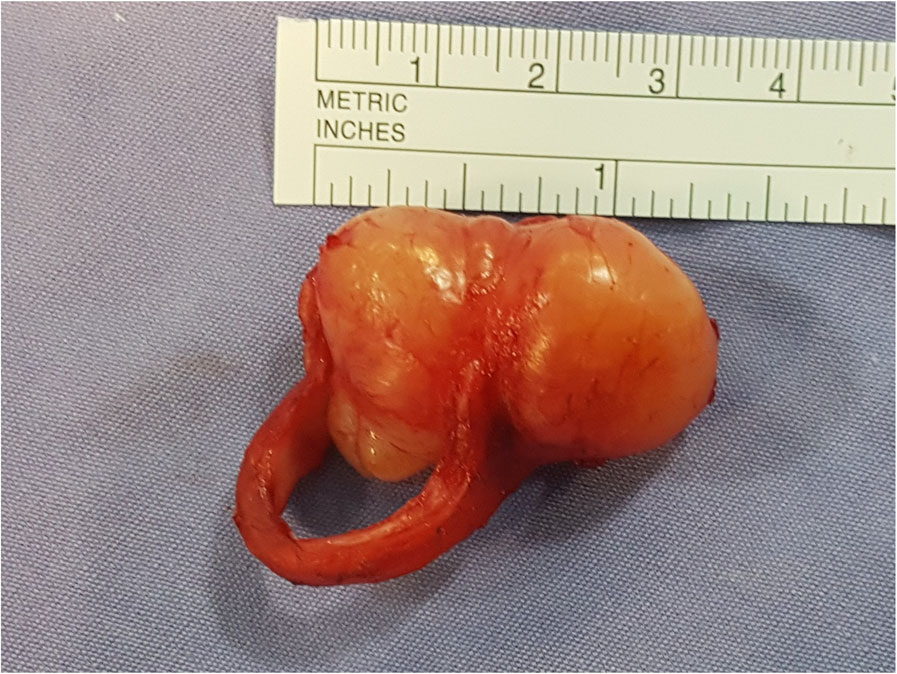
The resected dumbbell tumor (the other resected rings of the trachea where removed to illustrate the intraluminal extension of the tumor)

A 60-year-old man with history of type II diabetes mellitus, hypertension, ischemic heart disease, clear cell renal carcinoma (RCC), and recent low grade mucinous neoplasm of the appendix was referred from the urology clinic for evaluation of a scattered subcentimeter intraparenchymal pulmonary nodules found incidentally on follow up for his RCC. A bronchoscopy was performed by another service which revealed an incidental finding of a small posterior upper tracheal lesion. The mass was a less than two centimeter, mobile, irregular vascular tumor protruding from the posterior wall of the trachea and was three centimeters distal to the vocal cords causing partial obstruction. The lesion was excised using endotracheal laser resection. The histology showed primary tracheal schwannoma with positive resection margins. The intraparenchymal nodules were all less than 5 mm and follow up surveillance with low dose computer tomography was recommended. In November 2016 the patient developed a dry cough with occasional wheezes and his follow-up CT scan showed his tracheal tumor had recurred with extratracheal extension (Fig 1) and one of the intraparenchymal lung nodules had increased in size into 8 mm. Although the CT characteristics were suggestive of intraparenchymal granuloma, an integrated positron emission tomography with computer tomography (PET/CT) was ordered to better assess the intrapulmonary lesion and to assess any extrathoracic lesion. A bronchoscopy was also performed by the thoracic surgery service. The PET/CT showed a posterior upper tracheal lesion with extratracheal extension that was approximately 3X3 cm with an increased fludeoxyglucose (FDG) uptake with a standardized uptake value (SUV) of 6.5 (Fig 2). The intraparenchymal nodules were not active and there was no extrathoracic uptake. Because of this, follow up surveillance was recommended for the nodules. A bronchoscopy was performed which revealed a sessile smooth border tumor originating from the posterior wall of the trachea which obscured over 2/3 of the lumen. It extended three centimeters inferiorly from three centimeters distal to the vocal cords. The mucosal surface of the tumor was covered with small superficial blood vessels (Fig 3). Given his history, no biopsies were taken to avoid bleeding and potential for upper airway obstruction. One week later, a cervical approach was used to carry out the planned surgery. The patient’s neck and anterior chest were prepped and draped after the patient was placed in a supine position with hyper-extension of the neck by a sandbag behind the shoulders blades. An endotracheal flexometallic cuffed tube was inserted under bronchoscopic control and due to posterior extratracheal extension a nasogastric tube was also inserted. A transverse collar incision was made with subcutaneous and platysmal flaps were developed up to the level of the hyoid bone cranially and the sternal notch caudally. The strap muscles were dissected in the midline, the isthmus of the thyroid is transected and the dissection was continued to the pretracheal fascia. The inferior resection margin was defined by insertion of a needle under bronchoscopic guidance after pulling the endotracheal tube back by the anesthesiologist. The anterior aspect of the trachea up to the level of the bifurcation was mobilized well using a blunt finger mediastinal dissection. The tracheal wall was mobilized circumferentially at the planned inferior resection margin. Then the trachea was divided at the distal resection margin circumferentially after putting two anterolateral Vicryl 3–0 stay sutures in the distal airway and intubation the distal part of the airway across the surgical field using a sterile tubes and connections. The operative finding revealed a dumbbell tumor with half of the tumor being intratracheal and the other half extratracheal causing compression of the esophagus (Fig 4). The anesthesiologist pulled the previously inserted endotracheal tube back. The identified segment to be resected was around 2–3 cm and was mobilized including the extratracheal extension to the level of the proximal resection margin and then excised also after putting two anterolateral Vicryl 3/0 stay sutures in the proximal airway. Then the neck was taken out of extension and flexed with the help of a sandbag. The end-to-end anastomosis of the trachea is performed by using a combination of running posterior wall PDS 4/0 and interrupted anterior PDS 4/0 sutures. The proximal and distal trachea was approximated with the help of the Vicryl stay sutures to relieve tension as the anastomosis was created. The endotracheal tube is advanced and positioned distal to the anastomosis. After closure of the incision, a chin stitch “guard stitch” was placed and kept for 7 days to avoid accidental neck extension. The patient was extubated immediately postoperatively. His postoperative course was uneventful and the patient was discharged on the fourth post-operative day.

The macroscopic pathological examination showed a smooth dumbbell lesion originating from the posterior wall of the trachea. The microscopic examination illustrated a spindle cell neoplasm with well-differentiated Schwann cells. Histochemically, it had features consistent with cellular schwannoma: composed of predominantly Antoni A areas, positive for S100 protein expression diffusely, and the epithelial membrane antigen showed peripheral staining. There were no early or late post-operative complications and no recurrence of the tumor after a year follow-up. His follow-up is ongoing.

## Discussion

Primary tracheal neurogenic tumors are extremely rare. These tumors include mainly the benign peripheral nerve sheath tumors: neurofibroma and schwannoma [3, 4] and to date, there are only two reported cases of primary malignant endotracheal nerve sheath tumors [5, 6]. Schwannomas are extremely rare in the trachea, being more frequently reported in the lungs and bronchi [6].

Tracheal schwannoma was first reported in 1951 [7]. They are usually solitary, well-encapsulated lesions that are attached to the nerve sheath and sometimes covered with several small, discrete vessels [3, 6, 8, 9]. It is rarely associated with von Recklinghausen disease and also the malignant transformation is extremely rare [3, 8].

Kasahara et al. [10] proposed a classification of the pulmonary schwannomas. They divided the lesions into either central if the lesion is located in the trachea or in the proximal bronchi and can be seen by bronchoscopy, or peripheral when the lesions cannot be detected by bronchoscopy but can be detected by chest X-ray or computer tomography as a nodule. The central type is also divided into two subtypes: 1) tumors that exist only in the intraluminal space and 2) tumors that occur in both intraluminal and extraluminal spaces (combind type or dumbbell tumors).

Typically, most of the dumbbell shaped tumors of the trachea are malignant lesions with transmural extension of the mucosal lesion. However, this rule is not applicable in schwannoma because the lesion that is originating in the wall of the trachea and can be compressed by the cartilaginous rings either intraluminally or to the surrounding tissue of the trachea [8]. This particular shape of the pathology has been reported previously [4] and is also illustrated in our case.

In the published literature that describes this rare disease there are 4 successive prominent case reports with review of the literature in which the newer review includes the older reported cases [4–6, 8]. These reports are summarized in Table 1. Tang and colleagues describe, in detail, tracheal schwannomas in the pediatric age group that composes around one fifth of the reported cases [5].

**Table 1:** Selected case reports with review of the literature

	Horovitz AG et al. [8]	Righini CA et al. [4]	Tang LF et al. [5]	Xiahui GE et al. [6]
Years	1950–1983	1950–2003	1950–2005	1950–2013
Cases	13	23	34	51
Adults	11 (84.6%)	19 (82.6%)	27 (80%)	40 (78.4%)
F:M	7:5 (1 not specified)	14:4 (5 not specified)	18:13 (3 not specified)	30:20 (1 not specified)
Location in the trachea	distal > proximal > middle third	distal > proximal > middle third	NR	distal > proximal > middle third
Race	White > others	NR	NR	Asian > North American > European
Symptoms				
Common	Cough, wheeze, shortness of breath and hemoptysis	Upper airway obstruction with predominance of dyspnea	Airway obstruction symptoms like nonspecific cough, wheezing, and dyspnea.	Cough, wheezing and dyspnea
Uncommon	Fever and chest pain	Hemoptysis and chest infection	Hoarseness, hemoptysis, sudden severe dyspnea	Hemoptysis, hoarseness and chest pain
Signs	NR	NR	Pneumomediastinum, subcutaneous emphysema, and fever	Wheeze or Stridor 15 (29%)
Investigation	Fluoroscopy, Xeroradiographs and tomograms CT of the chest Tracheobronchoscopy	CT of the chest MRI (1 case) Tracheobronchoscopy	PFT CT of the chest MRI Tracheobronchoscopy	PFT CT of the chest Tracheobronchoscopy
Delay of the diagnosis	10–15 months	10–15 months	NR	17 months
Tumor size	NR	NR	NR	1–4 cm
Treatment				
Endoscopic resection (Laser, electronic snaring, APC, cryotherapy, endoscopic excision)	5 (38%)	NR	NR	19 (37%)
Surgical resection	8 (62%)	NR	NR	33 (71%) (29 primary treatment and 4 for relapse after endoscopic resection)
Recurrence				
Endoscopic resection	1 (20%)	1 (4%)	3(8.6%)	4 (21%)
Surgery	0 (0%)	0 (0.0%)	0 (0.0%)	0 (0%)
Malignant histology	NR	NR	2 (5.7%	2 (3.9%)
Complication	2	NR	3 (8.6%)	3 (6%)
	▪ Renal Failure ▪ Post-operative infection		▪ Renal Failure ▪ Post-operative infection ▪ Hypoxic brain damage	▪ Renal Failure ▪ Post-operative infection ▪ Hypoxic brain damage

*NR* not recorded, *CT* computer tomography, *MRI* Magnetic Resonance Image, *PFT* Pulmonary Function Test, *cm* centimeter

In the study that reviewed the literature between 1950 and 2013, only 51 cases of primary tracheal schwannoma were identified in the English literature [6]. In addition to these cases, a recent case has been also reported [11].

The clinical manifestations of intraluminal schwannomas of the trachea depend on the site, size, and the extent of obstruction produced by the tumor [6]. They can present with asthma like symptoms, symptoms of upper airway obstruction, and less frequently with hemoptysis and hoarseness [2, 6]. Due to the rarity of the primary tracheal schwannoma and its non-specific clinical manifestation, the average delay in diagnosis is 17 months from the onset of symptoms [6, 8].

Tracheal schwannoma is a disease of adults with female gender predilection [4–6, 8]. It most commonly found in the distal third of the trachea, followed by the proximal, and then middle thirds [3, 4, 6, 8].

The definitive diagnosis of primary tracheal schwannoma is usually made by tracheobronchoscopy with tissue biopsy taking in consideration all precautions that are needed in suspected upper airway obstruction. Additionally, multislice computerized tomography is used to delineate tumor size, site, and extratracheal extension [4–6]. Other adjunctive diagnostic method is the illustration of obstructive ventilatory defect on pulmonary function test as well as fixed upper airway obstruction in the flow volume loop [6, 12]. Although an integrated PET/CT was performed in our patient, the aim of it was to stratify the risk of the intraparenchymal nodules rather than to investigate the tracheal lesion. The elevation of FDG uptake in schwannomas and schwannomas with peritumoral lymphoid cuffs is known [13] and does not predict malignancy. Given that, we do not routinely recommend PET/CT for these tumors.

Tracheal schwannomas can be treated through multiple modalities: primary tracheal resection or endoscopic treatment including laser with or without a CO2, electrocautery snaring, argon plasma coagulation, cryotherapy, endoscopic excision, and microdebridement [6]. The choice of treatment should be influenced by the clinical presentation of the tumor (pedunculated vs. sessile), the risk of tracheal resection, and the presence or absence of an extratracheal component [14]. In our patient we elected to do tracheal resection as he had a recurrent sessile tumor with extratracheal extension and his preoperative risk was relatively low. In patients with a pedunculated lesion with no extratracheal extension or patients with high surgical risk, endoscopic resection is an option with bronchoscopic surveillance, understanding that recurrence occurs in nearly a quarter of patients [6, 14]. The time of recurrence is very variable [8, 15, 16] with a possibility of late recurrence (12 years in one case) [8]. Given that these tumors are slowly growing tumors it is preferable to have a scheduled follow up bronchoscopic surveillance annually. In the other group of patients, i.e. sessile tumors, having low surgical risk, or extratracheal extension, surgical resection is the best option for them with no reported cases of recurrence [6].

The prognosis for patients with schawnnomas that are either removed surgically or by endoscopic treatment methods is excellent [8].

## Conclusion

Primary endotracheal neurogenic tumors are extremely rare, but one should consider them in the differential diagnosis of persistent upper airway symptoms. To avoid recurrence, it is preferable to offer tracheal resection to low risk patients with sessile tumors or with extratracheal extension.

## Abbreviations

FDG: Fludeoxyglucose
PET/CT: Positron emission tomography with computer tomography
RCC: Clear cell renal carcinoma
SUV: Standardized uptake value
